# Associations between gestational age and childhood sleep: a national retrospective cohort study

**DOI:** 10.1186/s12916-022-02443-9

**Published:** 2022-08-08

**Authors:** Jiajun Lyu, John A. Groeger, Anna L. Barnett, Haifeng Li, Lei Wang, Jiajia Zhang, Wenchong Du, Jing Hua

**Affiliations:** 1 Shanghai Key Laboratory of Maternal Fetal Medicine, Shanghai First Maternity and Infant Hospital, School of Medicine, Tongji University, 2699 Gaoke Road, Shanghai, China; 2 NTU Psychology, Nottingham Trent University, Burton Street, Nottingham, NG1 4BU UK; 3 Centre for Psychological Research, Oxford Brookes University, Oxford, UK; 4 Department of Rehabilitation, The Children’s Hospital, Zhejiang University School of Medicine, National Clinical Research Center for Child Health, Zhejiang, China; 5 Maternal and Child Health Care Hospital of Yangzhou, Affiliated Hospital of Medical College Yangzhou University, Jiangsu, China

**Keywords:** Gestational age, Daily sleep duration, Sleep disorder, Children’s sleep habit questionnaire, Preschoolers

## Abstract

**Background:**

Both sleep quality and quantity are essential for normal brain development throughout childhood; however, the association between preterm birth and sleep problems in preschoolers is not yet clear, and the effects of gestational age across the full range from preterm to post-term have not been examined. Our study investigated the sleep outcomes of children born at very-preterm (<31 weeks), moderate-preterm (32–33 weeks), late-preterm (34–36 weeks), early-term (37–38 weeks), full-term (39–40 weeks), late-term (41 weeks) and post-term (>41 weeks).

**Methods:**

A national retrospective cohort study was conducted with 114,311 children aged 3–5 years old in China. Children’s daily sleep hours and pediatric sleep disorders defined by the Children’s Sleep Habits Questionnaire (CSHQ) were reported by parents. Linear regressions and logistic regression models were applied to examine gestational age at birth with the sleep outcomes of children.

**Results:**

Compared with full-term children, a significantly higher CSHQ score, and hence worse sleep, was observed in very-preterm (*β* = 1.827), moderate-preterm (*β* = 1.409), late-preterm (*β* = 0.832), early-term (*β* = 0.233) and post-term (*β* = 0.831) children, all *p*<0.001. The association of pediatric sleep disorder (i.e. CSHQ scores>41) was also seen in very-preterm (adjusted odds ratio [AOR] = 1.287 95% confidence interval [CI] (1.157, 1.433)), moderate-preterm (AOR = 1.249 95% CI (1.110, 1.405)), late-preterm (AOR = 1.111 95% CI (1.052, 1.174)) and post-term (AOR = 1.139 95% CI (1.061, 1.222)), all *p*<0.001. Shorter sleep duration was also found in very-preterm (*β* = −0.303), moderate-preterm (*β* = −0.282), late-preterm (*β* = −0.201), early-term (*β* = −0.068) and post-term (*β* = −0.110) compared with full-term children, all *p*<0.01. Preterm and post-term-born children had different sleep profiles as suggested by subscales of the CSHQ.

**Conclusions:**

Every degree of premature, early-term and post-term birth, compared to full-term, has an association with sleep disorders and shortened daily sleep duration. Preterm, early-term, and post-term should therefore all be monitored with an increased threat of sleep disorder that requires long-term monitoring for adverse sleep outcomes in preschoolers.

**Supplementary Information:**

The online version contains supplementary material available at 10.1186/s12916-022-02443-9.

## Background

It is well established that both sleep quality and quantity are essential for normal brain development throughout childhood, particularly for cognitive functions [1]. However, sleep problems are relatively common among young children, and according to parent reports, it has been suggested that approximately 20–30% of young children have various sleep problems [2]. In preterm infants younger than 1 year old, sleep problems are thought to be even more prevalent [3, 4].

However, the exact relationship between preterm birth and sleep problems beyond infancy is not yet clear. Studies have suggested that school-aged preterm children had different sleep patterns compared to full-term children, such as having earlier bedtimes and earlier wake times [5–7], but had no difference in overall sleep duration [6, 7]. Preterm children have been reported to have lower sleep quality, including more nocturnal awakenings [8, 9], and consistent with this more “shallow” and less “deep” non-rapid eye movement sleep [9]. It has been suggested that irreversible adverse factors related to preterm birth, such as brain injury, altered brain maturation, and respiratory problems may precipitate poor sleep. Furthermore, a range of parental factors related to preterm childcaring may also play a role. For example, increased parental concern about preterm children may be linked to earlier bedtimes, which may contribute to the different sleep outcomes of preterm children beyond infancy [10]. However, given the very limited studies available in the literature, variation in the degrees of prematurity and sample size makes it difficult to draw any clear conclusions about the sleep outcomes of preterm children beyond infancy.

Moreover, to our knowledge, no study has been conducted to date on the sleep outcomes of post-term-born children (>41 weeks). Studies have reported that post-term birth can negatively affect children’s short-term and long-term health outcomes [11–15]. Post-term birth can increase the risk of neonatal encephalopathy and death during the first year of life [16]. It has also been reported that, with respect to longer-term effects, post-term birth increases the risks of cognitive impairments, severe mental disorders, neuropsychological disorders, and other behavioural and emotional problems in early childhood [17–20]. Post-term delivery often has a higher risk for perinatal problems such as prolonged labour that can cause a perinatal lack of oxygen [21] and uteroplacental insufficiency [15]. These risk factors may predispose infants to abnormal brain development and respiratory problems [22] which may lead in turn to sleep problems in post-term children.

Therefore, in the current study, we used a retrospective cohort study design to systematically examine the effect of gestational age on sleep outcomes with a large sample of urban Chinese children. We hypothesized that compared with full-term children (39–40 weeks), that born very-preterm (<31 weeks), moderate-preterm (32–33 weeks), late-preterm (34–36 weeks), early-term (37–38 weeks) and post-term (>41 weeks) all had a higher incidence of sleep disorders and altered sleep outcomes.

## Methods

### Study design and participants

The present study was part of the Chinese National Cohort of Motor Development (CNCMD), which was designed to explore the neurobehavioral development and other health outcomes (including sleep health, cognition and language development) in Chinese preschool children [23]. A stratified cluster sampling plan was used to ensure that the participants included in the current study were representative of the Chinese population. The China 2018–2019 National Census provided the basis for the stratification by geographic region, age, sex, and socioeconomic status (SES). Ethnic information was not collected because more than 99% of the population in the targeted regions were Han according to the National Census. The government-supported maternity and children’s healthcare centre in each city were selected to invite their local kindergartens to participate in the study. Class teachers were responsible for distributing the notification to parents to complete an online questionnaire. Names and phone numbers of the researchers were provided in case the parents or teachers had queries about the study or about how to respond to the questionnaires. We used an electronic online questionnaire system to enhance the quality of the data by allowing the inclusion of pop-up instructions, error messages, links to further information and to set conditions to ensure participants could not skip questions. A data coordination centre was established to take charge of establishing, managing and maintaining the database, coordinating among health centres.

It is a normal practice for parents to keep in touch with their children’s nursery via smart devices in China, including all of the kindergartens involved in the current study. It was therefore assumed that all of the parents in the current study had relatively high proficiency in online questionnaire completion. All parents gave consent before starting to take part in the study.

**Fig. 1:**
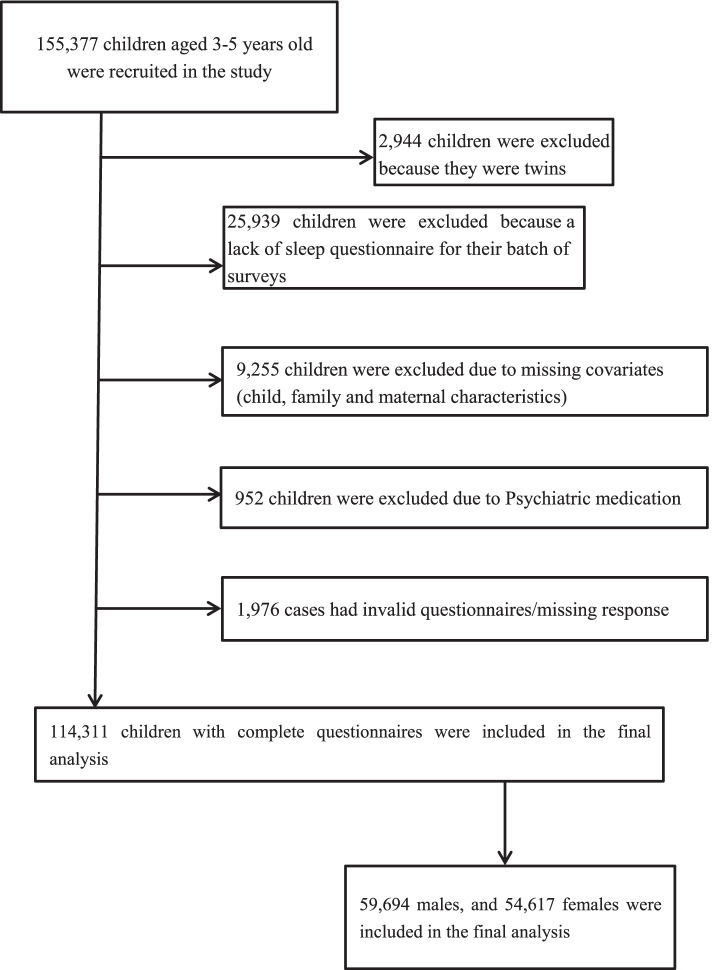
Flowchart of the study population. Legend: Detailed presentation of the inclusion and exclusion criteria of the study participant selection process and how the final number of the study cohort was established

From April 1, 2018, to December 31, 2019, a total of 155,377 children aged 3–5 years old from 2403 nurseries in 551 cities in China were recruited for the study. Children were excluded from the study if they had severe visual, hearing, intellectual impairments, cerebral palsy or other severe developmental disorders including autism spectrum disorder (ASD) who were required to receive special education needs and to attend special education schools/nurseries according to the local regulations. Only mainstream schools/nurseries were included in the study; this is the regular provision which excludes special education schools/nurseries. Children with any of the following conditions that may affect the accuracy of the information collected with the questionnaire were excluded from the study: (1) death of the mother; (2) illiterate parents; (3) children taking certain medications with known effects on sleep (such as aspirin, ritalin, amphetamine, caffeine, diazepam, phenobarbital) longer than 1 week at the time of the survey completion date [24–27]. Children who were twins or had missing covariates were also excluded. Parents of 25,939 children chose not to participate or left the questionnaire before fully completing it. In all, 114,311 children were included in the final analysis (Fig. 1).

The study was approved by the Ethics Committee of Shanghai First Maternity and Infant Hospital (KS18156). All information acquired was kept confidential and was only accessible by the researchers.

### Measures

#### Outcome variables

Sleep duration and sleep disorders of the children were measured with the Children’s Sleep Habits Questionnaire (CSHQ) [28]. There are 33 parent-rated items in the questionnaire that assesses the frequency of behaviours associated with common pediatric sleep difficulties. The CSHQ instructs parents to rate the frequency with which their child has displayed various sleep behaviours in a typical week during the previous 4 weeks. Ratings are combined to create eight subscales that relate to common sleep problems in children: Bedtime Resistance, Sleep Onset Delay, Sleep Duration, Sleep Anxiety, Night Wakings, Parasomnias, Sleep Disordered Breathing, and Daytime Sleepiness. Finally, all ratings are summed to create a total sleep disturbance index. A higher CSHQ score indicates more sleep difficulties, and a score of over 41 indicates a pediatric sleep disorder [28]. The CSHQ has been shown to be a valid and reliable measurement when it is used with Chinese children [29].

Two extra questions were also included in the parent questionnaire to collect information on the exact daily sleep hours of the children: “How many hours does your child sleep during the weekdays?” and “How many hours does your child sleep at the weekends (including daytime nap)?” Daily sleep hours were then calculated as the value of 5/7×Sleep hours during weekdays+2/7×Sleep hours at the weekends [30]. Daytime nap during the weekdays was not asked for because all of the recruited children attended nursery full-time and a standard daytime nap for the same duration was part of the daily routine in all nurseries.

#### Independent variables

Gestational age at birth was obtained from the mother’s medical records, which was based on ultrasound examination and date of last menstrual period (LMP). Following the literature [31], seven categories of gestational ages were decided: very-preterm (<31 weeks), moderate-preterm (32–33 weeks), late-preterm (34–36 weeks), early-term (37–38 weeks), completely full-term (39–40 weeks), late-term (41 weeks) and post-term (>41 weeks).

#### Covariates

We included a range of family, child and maternal characteristics as covariates according to the literature: (1). Child characteristics included the child’s age, sex, child body mass index (BMI), right-handedness, eyesight, birth weight, delivery mode, newborn intensive care unit (NICU) admission and developmental disorders (attention deficit syndrome, attention deficit and hyperactivity disorder, learning disabilities). Body mass index (BMI) is an indicator of obesity that is based on height and weight (BMI = weight(kg)/height(m)). Eyesight was grouped into normal and abnormal (including myopia, hyperopia, astigmatism). Co-sleeping was identified with a particular question: “How often does your child sleep in the parent(s)/caregiver(s) bed at night?” The answers were (1) usually, 5 to 7 nights per week; (2) sometimes, 2 to 4 nights per week; (3) rarely, 0 to 1 night per week. In this study, we defined co-sleeping as bed-sharing that occurred 5 to 7 nights per week [32]. (2) Family characteristics included the following variables: higher education of mother and father (yes vs. no); mother and father’s employment status (employed vs. unemployed); family annual per capita income; the number of children in the family (one vs. two or more); and family structure (single-family, nuclear family and extended family). The “single-family” means the child lives with one of his/her parents; the “nuclear family” refers to the child living only with his/her parents; and the “extended family” refers to the child living with his/her parents and grandparents, which is a traditional family structure in China. (3) Maternal health characteristics included the following variables: maternal age at delivery (<30, 30–34 and ≥35 years); smoking or passive smoking during pregnancy; and maternal complications during pregnancy. Maternal complications were defined according to the International Classification of Diseases, Revision 10 (ICD 10) [33]. The classification is defined as having one of the following maternal complications during pregnancy: gestational diabetes, hypertensive disorders, vaginal bleeding during pregnancy, risk of miscarriage, use of antibiotics, use of fertility drugs, intrauterine distress and foetal asphyxia.

### Statistical analysis

Differences in the child, family and maternal health characteristics by sex and gestational age categories were analysed using independent *t*-tests, one-way analysis of variance (ANOVA) and chi-squared tests. Based on the previous literature [34–38] and our exploratory analysis, the summarized directed acyclic graph (DAG) between gestational age, daily sleep hours and CSHQ scores (Additional file 1: Figure S1) was generated by using a web-based tool DAGitty (www.dagitty.net). As shown in the DAGs, maternal characteristics and family characteristics as confounders; age, gender, eyesight and co-sleep were considered as competing exposures. Birth weight, BMI, NICU admission, other developmental disorder, handedness and delivery mode were considered as mediators. The distribution of sleep hours and CSHQ scores were relatively symmetric, and multivariable linear regression analysis was conducted to examine the associations of gestational age with daily sleep hours and the CSHQ scores when adjusting for all confounders and competing exposures, while the mediators as the variables on the causal pathway were not included in the adjusted model [39]. Logistic regression analyses were then conducted to examine the associations between different gestational groups and sleep disorders when adjusting for all confounders and competing exposures, while mediators were not included in the adjusted model.

A statistical significance level was set at a *p*-value <0.05 (two-tailed). To correct for multiple testing, the Benjamini-Hochberg false discovery rate method was used to decrease the probability of false positives [40]. All analyses were performed with the Statistical Package for the Social Sciences (SPSS) (IBM-SPSS Statistics v24.0, Inc Chicago, IL) and R version 3.5.3.

## Results

### Sample demographic characteristics

A total of 114,311 children aged 4.40±0.79 years old were enrolled in the final analysis. Among all the children, 54,617 (47.8%) were girls and 59,694 (52.2%) were boys. A total of 2379 (2.08%) were very-preterm births; 1893 (1.66%) were moderate-preterm births; 10,238 (9.96%) were late-preterm births; 29,179 (25.53%) were early-term births; 58,043 (50.78%) were full-term births; 7016 (6.14%) were late-term births; 5563 (4.87%) were post-term births (Table 1). Co-sleeping was common, and 82.02% of the children shared a bed with their parents (Table 2). The average daily sleep hours is 10.715±2.693 (Table 1), and 87,773 children (76.78%) were reported to have sleep disorders (Table 3). The prevalence of sleep disorders was 81.21%, 80.67%, 78.62.%, 76.58%, 76.02%, 76.82% and 79.17%, in very-preterm, moderate-preterm, late-preterm, early-term, full-term, late-term and post-term births, respectively (Table 3). The child, family and maternal health during pregnancy characteristics in the study population are shown in Table 2.

**Table 1:** The description of daily sleep hours and CSHQ score stratified by age and gestational in preschoolers (*n* = 114,311)

	***N*, %**	**Daily sleep hours** **(means, SD)**	**CSHQ score** **(means, SD)**
**Total**	114311, 100.00%	10.715±2.693	46.706±7.576
Very-preterm (<31W)	2379, 2.08%	10.409±3.135	48.380±8.787
Moderate-preterm (32–33W)	1893, 1.66%	10.433±2.951	47.940±8.556
Late-preterm (34–36W)	10238, 8.96%	10.505±2.885	47.340±8.137
Early-term (37–38W)	29179, 25.53%	10.689±2.699	46.664±7.582
Full-term (39–40W)	58043, 50.78%	10.780±2.616	46.464±7.355
Late-term (41–41W)	7016, 6.14%	10.852±2.529	46.539±7.124
Post-term (>41W)	5563, 4.87%	10.614±2.928	47.365±8.138
**3 years old**	37823, 100.00%	46.666±7.052	46.592±6.863
Very-preterm (<31W)	726, 1.92%	10.339±3.231	48.906±8.893
Moderate-preterm (32–33W)	530, 1.40%	10.432±2.97	48.502±8.75
Late-preterm (34–36W)	3022, 7.99%	10.623±2.826	47.254±7.500
Early-term (37–38W)	9824, 25.97%	10.802±2.639	46.846±7.293
Full-term (39–40W)	19647, 51.94%	10.879±2.552	46.666±7.052
Late-term (41–41W)	2435, 6.44%	10.851±2.499	47.689±7.942
Post-term (>41W)	1639, 4.33%	10.664±2.853	46.846±7.293
**4 years old**	43847, 100.00%	10.708±2.685	46.781±7.545
Very-preterm(<31W)	938, 2.14%	10.451±3.102	48.263±8.404
Moderate-preterm (32–33W)	768, 1.75%	10.530±2.918	47.965±8.281
Late-preterm (34–36W)	3890, 8.87%	10.530±2.857	47.511±8.096
Early-term (37–38W)	10997, 25.08%	10.654±2.711	46.748±7.544
Full-term (39–40W)	22276, 50.80%	10.766±2.609	46.493±7.303
Late-term (41–41W)	2779, 6.34%	10.654±2.711	46.800±7.283
Post-term (>41W)	2199, 5.02%	10.885±2.459	47.514±8.343
**5 years old**	32641, 100.00%	10.615±2.768	46.422±7.968
Very-preterm (<31W)	715, 2.19%	10.424±3.082	48.000±9.152
Moderate-preterm (32–33W)	595, 1.82%	10.308±2.975	47.398±8.711
Late-preterm (34–36W)	3326, 10.19%	10.368±2.965	47.222±8.720
Early-term (37–38W)	8358, 25.61%	10.604±2.748	46.339±7.948
Full-term (39–40W)	16120, 49.39%	10.678±2.699	46.177±7.770
Late-term (41–41W)	1802, 5.52%	10.802±2.673	46.065±7.203
Post-term (>41W)	1725, 5.28%	10.534±2.953	46.957±8.124

Daily sleep hours = 5/7*sleep hours on weekdays +2/7*sleep hours at the weekends

*CSHQ* Children’s Sleep Habit Questionnaire, *SD* standard deviation

**Table 2:** The child and family characteristics in the study population (*n* = 114,311)

**Characteristics**	Total	Sex		***p***	Gestational age at birth							***p***
	(*n* = 114,311)	Male	Female		Very-preterm	Moderate-preterm	Preterm	Early-term	Full-term	Late-term	Post-term	
		(*n* = 59,694)	(*n* = 54,617)		<31W	32–33W	34–36W	37–38W	39–40W	41–41W	>41W	
					(*n* = 2379)	(*n* = 1893)	(*n* = 10,238)	(*n* = 29,179)	(*n* = 58,043)	(*n* = 7016)	(*n* = 5563)	
**Child characteristics**												
Children’s age (M, SD)	4.408, 0.798	4.413, 0.797	4.403, 0.800	0.0423	4.446, 0.790	4.501, 0.781	4.490, 0.801	4.411, 0.804	4.389, 0.797	4.347, 0.782	4.472, 0.789	<0.001
Gender (*n*%)												<0.001
Male	59,694, 52.221	59,694, 100.000	0, 0.000		1268, 53.3	1014, 53.566	5568, 54.386	16,010, 54.868	29,670, 51.117	3373, 48.076	2791, 50.171	
Female	54,617, 47.779	0, 0.000	54,617, 100.000		1111, 46.7	879, 46.434	4670, 45.614	13,169, 45.132	28,373, 48.883	3643, 51.924	2772, 49.829	
BMI (M, SD)	15.602, 1.607	15.754, 1.622	15.435, 1.574	<0.001	15.660, 1.709	15.661, 1.666	15.660, 1.702	15.609, 1.617	15.576, 1.576	15.582, 1.574	15.698, 1.669	<0.001
Right-handedness (*n*%)				<0.001								0.007
No	8187, 7.162	4763, 7.979	3424, 6.269		212, 8.911	148, 7.818	763, 7.453	2103, 7.207	4039, 6.959	520, 7.412	402, 7.226	
Yes	106,124, 92.838	54,931, 92.021	51,193, 93.731		2167, 91.089	1745, 92.182	9475, 92.547	27,076, 92.793	54,004, 93.041	6496, 92.588	5161, 92.774	
Eyesight (*n*%)				0.506								0.147
Normal	103,199, 90.279	53,858, 90.223	49,341, 90.34		2133, 89.66	1691, 89.329	9202, 89.881	26,353, 90.315	52,463, 90.386	6302, 89.823	5055, 90.868	
Abnormal	11,112, 9.721	5836, 9.777	5276, 9.66		246, 10.34	202, 10.671	1036, 10.119	2826, 9.685	5580, 9.614	714, 10.177	508, 9.132	
Birth weight (*n*%)				<0.001								<0.001
<2500g	3700, 3.237	1727, 2.893	1973, 3.612		365, 15.343	383, 20.232	1019, 9.953	925, 3.17	808, 1.392	93, 1.326	107, 1.923	
≥2500g	110,611, 96.763	57,967, 97.107	52,644, 96.388		2014, 84.657	1510, 79.768	9219, 90.047	28,254, 96.83	57,235, 98.608	6923, 98.674	5456, 98.077	
Delivery mode				<0.001								<0.001
Vaginal delivery	59,625, 52.160	30,421, 50.962	29,204, 53.471		1208, 50.778	928, 49.023	5070, 49.521	13,925, 47.723	31,639, 54.51	3893, 55.487	2962, 53.245	
Delivery with caesarean section	54,686, 47.840	29,273, 49.038	25,413, 46.529		1171, 49.222	965, 50.977	5168, 50.479	15,254, 52.277	26,404, 45.49	3123, 44.513	2601, 46.755	
NICU admission				<0.001								<0.001
No	102,833, 89.959	53,301, 89.290	49,532, 90.69		1891, 79.487	1399, 73.904	8206, 80.152	26,290, 90.099	53,501, 92.175	6420, 91.505	5126, 92.145	
Yes	11,478, 10.041	6393, 10.710	5085, 9.31		488, 20.513	494, 26.096	2032, 19.848	2889, 9.901	4542, 7.825	596, 8.495	437, 7.855	
Other developmental disorders				<0.001								<0.001
No	113,565, 99.347	59,244, 99.246	54,321, 99.458		2359, 99.159	1867, 98.627	10174, 99.375	28,971, 99.287	57,702, 99.413	6977, 99.444	5515, 99.137	
Yes	746, 0.653	450, 0.754	296, 0.542		20, 0.841	26, 1.373	64, 0.625	208, 0.713	341, 0.587	39, 0.556	48, 0.863	
Co-sleeping				0.427								0.142
Yes	93,755, 82.020	48,941, 81.900	44,814, 82.000		1935, 81.337	1530, 80.824	8395, 81.998	23,914, 81.956	47,717, 82.21	5754, 82.013	4510, 81.071	
No	20,556, 17.980	10,753, 18,100	9803, 18.000		444, 18.663	363, 19.176	1843, 18.002	5265, 18.044	10,326, 17.79	1262, 17.987	1053, 18.929	
**Family characteristics**												
Higher education of mother (*n*%)				0.00195								<0.001
No	51,393, 44.959	27,098, 45.395	24,295, 44.482		1086, 45.649	862, 45.536	4578, 44.716	13,540, 46.403	24,070, 41.469	2929, 41.747	2870, 51.591	
Yes	62,918, 55.041	32,596, 54.605	30,322, 55.518		1293, 54.351	1031, 54.464	5660, 55.284	15,639, 53.597	33,973, 58.531	4087, 58.253	2693, 48.409	
Higher education of father (*n*%)				0.00498								<0.001
No	52,335, 45.783	27,566, 46.179	24,769, 45.35		1061, 44.599	851, 44.955	4496, 43.915	13,709, 46.982	24,759, 42.656	2940, 41.904	2825, 50.782	
Yes	61,976, 54.217	32,128, 53.821	29,848, 54.65		1318, 55.401	1042, 55.045	5742, 56.085	15,470, 53.018	33,284, 57.344	4076, 58.096	2738, 49.218	
Mother’s occupation (*n*%)				0.00255								0.013
Employed	71,455, 62.509	37,561, 62.923	33,894, 62.058		1505, 63.262	1165, 61.543	6231, 60.861	18,247, 62.535	36,361, 62.645	4439, 63.27	3507, 63.042	
Unemployed	42,856, 37.491	22,133, 37.077	20,723, 37.942		874, 36.738	728, 38.457	4007, 39.139	10,932, 37.465	21,682, 37.355	2577, 36.73	2056, 36.958	
Father’s occupation (*n*%)				0.249								<0.001
Employed	90,554, 79.217	47,367, 79.35	43,187, 79.072		1834, 77.091	1452, 76.704	7755, 75.747	23,101, 79.17	46,381, 79.908	5655, 80.601	4376, 78.663	
Unemployed	23,757, 20.783	12,327, 20.65	11,430, 20.928		545, 22.909	441, 23.296	2483, 24.253	6078, 20.83	11,662, 20.092	1361, 19.399	1187, 21.337	
Family annual per capita income (RMB) b(*n*%)				0.0326								<0.001
Below	21,988, 19.235	11,340, 18.997	10,648, 19.496		526, 22.11	431, 22.768	2371, 23.159	5418, 18.568	10,683, 18.405	1296, 18.472	1263, 22.704	
Above or equal to	92,323, 80.765	48,354, 81.003	43,969, 80.504		1853, 77.89	1462, 77.232	7867, 76.841	23,761, 81.432	47,360, 81.595	5720, 81.528	4300, 77.296	
Family structure (*n*%)				0.0395								
												<0.001
Single families	2807, 2.456	1412, 2.365	1395, 2.554		89, 3.741	59, 3.117	340, 3.321	748, 2.563	1242, 2.140	155, 2.209	174, 3.128	
Nuclear families	70,142, 61.361	36,724, 61.520	33,418, 61.186		1544, 64.901	1225, 64.712	6581, 64.28	17,989, 61.651	35,235, 60.705	4058, 57.839	3510, 63.095	
Extended families	41,362, 36.184	21,558, 36.114	19,804, 36.260		746, 31.358	609, 32.171	3317, 32.399	10,442, 35.786	21,566, 37.155	2803, 39.952	1879, 33.777	
The number of children in the family (*n*%)				<0.001								<0.001
One	64,273, 56.226	34,319, 57.492	29,954, 54.844		1390, 58.428	1125, 59.429	5616, 54.854	15,516, 53.175	33,105, 57.035	4201, 59.877	3320, 59.680	
Two or more	50,038, 43.774	25,375, 42.508	24,663, 45.156		989, 41.572	768, 40.571	4622, 45.146	13,663, 46.825	24,938, 42.965	2815, 40.123	2243, 40.320	
**Maternal health characteristics**												
Maternal age at delivery (*n*%)				0.414								<0.001
<30	84,676, 74.075	44,158, 73.974	40,518, 74.186		1766, 74.233	1354, 71.527	7068, 69.037	20,500, 70.256	44,021, 75.842	5686, 81.043	4281, 76.955	
30–34	21,956, 19.207	11,582, 19.402	10,374, 18.994		417, 17.528	367, 19.387	2224, 21.723	6293, 21.567	10,666, 18.376	1049, 14.952	940, 16.897	
≥35	7679, 6.718	3954, 6.624	3725, 6.820		196, 8.239	172, 9.086	946, 9.24	2386, 8.177	3356, 5.782	281, 4.005	342, 6.148	
Smoking or passive smoking during pregnancy (*n*%)				0.685								<0.001
No	82,461, 72.137	43,031, 72.086	39,430, 72.194		1677, 70.492	1336, 70.576	7154, 69.877	21,197, 72.645	42,124, 72.574	5019, 71.536	3954, 71.077	
Yes	31,850, 27.863	16,663, 27.914	15,187, 27.806		702, 29.508	557, 29.424	3084, 30.123	7982, 27.355	15,919, 27.426	1997, 28.464	1609, 28.923	
Maternal complications during pregnancy c(*n*%)				0.147								<0.001
No	108,934, 95.296	56,938, 95.383	51,996, 95.201		2242, 94.241	1755, 92.71	9612, 93.886	27,515, 94.297	55,634, 95.85	6783, 96.679	5393, 96.944	
Yes	5377, 4.704	2756, 4.617	2621, 4.799		137, 5.759	138, 7.29	626, 6.114	1664, 5.703	2409, 4.15	233, 3.321	170, 3.056	

Other developmental disorders included attention deficit syndrome, attention deficit and hyperactivity disorder, learning disabilities

**Table 3:** The age-specific association between gestational age and sleep disorder in preschoolers (*n*=114,311)

	**Sleep disorder (CSHQ>41)**							
	***N*, %**		**Crude OR (95% CI)**	***p***	p*	**Adjusted OR**^**a**^** (95% CI)**	***p***	p*
	**Yes**	**No**						
**Total**	87,773, 76.78%	26,538, 23.22%						
Very-preterm(<31W)	1932, 81.21%	447, 18.79%	**1.322 (1.188, 1.47)**	**<0.001**	**<0.001**	**1.287 (1.157, 1.433)**	**<0.001**	**<0.001**
Moderate-preterm(32–33W)	1527, 80.67%	366, 19.33%	**1.276 (1.135, 1.435)**	**<0.001**	**<0.001**	**1.249 (1.110, 1.405)**	**<0.001**	**<0.001**
Late-preterm (34–36W)	8049, 78.62%	2189, 21.38%	**1.125 (1.065, 1.188)**	**<0.001**	**<0.001**	**1.111 (1.052, 1.174)**	**<0.001**	**<0.001**
Early-term (37–38W)	22,345, 76.58%	6834, 23.42%	0.97 (0.938, 1.002)	0.069	0.828	**0.958 (0.927, 0.991)**	**0.012**	**0.014**
Full-term (39–40W)	44,126, 76.02%	13917, 23.98%	Reference			Reference		
Late-term (41–41W)	5390, 76.82%	1626, 23.18%	1.014 (0.953, 1.078)	0.663	0.663	0.976 (0.917, 1.039)	0.451	0.451
Post-term (>41W)	4404, 79.17%	1159, 20.83%	**1.162 (1.083, 1.247)**	**<0.001**	**<0.001**	**1.139 (1.061, 1.222)**	**<0.001**	**<0.001**
**3 years old**	29,658, 78.40%	8165, 21.60%						
Very-preterm(<31W)	599, 82.51%	127, 17.49%	**1.308 (1.074, 1.594)**	**0.008**	**0.048**	1.267 (1.039, 1.545)	**0.019**	0.114
Moderate-preterm(32–33W)	435, 82.08%	95, 17.92%	**1.27 (1.012, 1.594)**	0.039	0.078	1.24 (0.987, 1.558)	0.065	0.195
Late-preterm (34–36W)	2377, 78.66%	645, 21.34%	1.022 (0.925, 1.129)	0.667	0.667	0.996 (0.901, 1.100)	0.930	0.930
Early-term (37–38W)	7691, 78.29%	2133, 21.71%	0.981 (0.925, 1.04)	0.523	0.667	0.968 (0.912, 1.027)	0.277	0.415
Full-term (39–40W)	15,317, 77.96%	4330, 22.04%	Reference			Reference		
Late-term (41–41W)	1918, 78.77%	517, 21.23%	1.029 (0.923, 1.147)	0.606	0.667	0.985 (0.883, 1.099)	0.787	0.930
Post-term (>41W)	1321, 80.60%	318, 19.40%	1.152 (1.01, 1.314)*	0.035	0.078	1.111 (0.974, 1.269)	0.118	0.236
**4 years old**	33,962, 77.46%	9885, 22.54%						
Very-preterm(<31W)	772, 82.30%	166, 17.70%	**1.352 (1.137, 1.608)**	**0.001**	**0.002**	**1.295 (1.088, 1.542)**	**0.004**	**0.008**
Moderate-preterm(32–33W)	636, 82.81%	132, 17.19%	**1.401 (1.155, 1.699)**	**0.001**	**0.002**	**1.35 (1.112, 1.639)**	**0.002**	**0.006**
Late-preterm (34–36W)	3120, 80.21%	770, 19.79%	**1.178 (1.076, 1.29)**	**<0.001**	**<0.001**	**1.155 (1.055, 1.266)**	**0.002**	**0.006**
Early-term (37–38W)	8520, 77.48%	2477, 22.52%	**0.942 (0.892, 0.995)**	**0.031**	**0.0372**	**0.931 (0.882, 0.984)**	**0.011**	**0.016**
Full-term (39–40W)	17,022, 76.41%	5254, 23.59%	Reference			Reference		
Late-term (41–41W)	2140, 77.01%	639, 22.99%	0.974 (0.882, 1.075)	0.597	0.597	0.945 (0.855, 1.045)	0.269	0.269
Post-term (>41W)	1752, 79.67%	447, 20.33%	**1.139 (1.018, 1.276)**	**0.024**	**0.036**	1.097 (0.979, 1.229)	0.111	0.133
**5 years old**	24,153, 74.00%	8488, 26.00%						
Very-preterm(<31W)	561, 78.46%	154, 21.54%	**1.321 (1.098, 1.589)**	**0.003**	**0.006**	**1.293 (1.074, 1.557)**	**0.007**	**0.014**
Moderate-preterm(32–33W)	456, 76.64%	139, 23.36%	1.189 (0.978, 1.447)	0.083	0.1245	1.155 (0.949, 1.407)	0.151	0.226
Late-preterm (34–36W)	2552, 76.73%	774, 23.27%	**1.195 (1.088, 1.313)**	**<0.001**	**<0.001**	**1.172 (1.066, 1.288)**	**0.001**	**0.006**
Early-term (37–38W)	6134, 73.39%	2224, 26.61%	0.986 (0.929, 1.047)	0.651	0.651	0.979 (0.922, 1.04)	0.491	0.589
Full-term (39–40W)	11,787, 73.12%	4333, 26.88%	Reference			Reference		
Late-term (41–41W)	1332, 73.92%	470, 26.08%	1.028 (0.915, 1.154)	0.646	0.651	1.002 (0.892, 1.126)	0.975	0.975
Post-term (>41W)	1331, 77.16%	394, 22.84%	**1.225 (1.084, 1.384)**	**0.001**	**0.003**	**1.209 (1.069, 1.368)**	**0.002**	**0.006**

*CSHQ* Children’s Sleep Habit Questionnaire, *CI* confidence interval, *OR* odds ratio

^a^Adjusted for age, gender, eyesight, co-sleep, maternal characteristics and family characteristics

p* value corrected after multiple testing

^b^Statistically significant results (*p* < 0.05) are in bold

### Association of gestational age with childhood daily sleep hours and sleep disorder

Compared with completely full-term-born children, higher CSHQ scores were observed in very-preterm, moderate-preterm, late-preterm, early-term and post-term categories. Very-preterm, moderate-preterm, late-preterm and post-term birth were associated with higher CSHQ scores (*β* = 1.827, 1.409, 0.832, and 0.831 respectively, each *p<*0.001, *p correction*<0.001, Table 4), and very-preterm, moderate-preterm, late-preterm, early-term and post-term categories were associated with shorter daily sleep hours (*β* = −0.303, −0.282, −0.201, −0.068 and −0.110 respectively, each *p*<0.01, *p correction* <0.01, Table 4) after controlling for confounders and competing exposures. These positive associations of gestational age with the CSHQ scores were also found within all three age groups (Table 4). The significance of post-term with daily sleep hours was only shown in the 3-year-old group (Table 4).

**Table 4:** The age-specific association between gestational age and score with daily sleep hours and CSHQ score in preschoolers(*n*=114,311)

	**Daily sleep hours (10.715±2.693)**						**CSHQ score (46.706±7.576)**					
	**Crude *β*** **(95% CI)**	***p***	p*	**Adjusted *β***^**a**^ **(95% CI)**	***p***	p*	**Crude *β* (95% CI)**	***p***	p*	**Adjusted *β***^**a**^ **(95% CI)**	***p***	p*
**Total**												
Very-preterm (<31W)	**−0.371 (−0.482, −0.261)**	**<0.001**	**<0.001**	**−0.303 (−0.413, −0.193)**	**<0.001**	**<0.001**	**1.917 (1.606, 2.227)**	**<0.001**	**<0.001**	**1.827 (1.518, 2.136)**	**<0.001**	**<0.001**
Moderate-preterm (32–33W)	**−0.348 (−0.471, −0.224)**	**<0.001**	**<0.001**	**−0.282 (−0.405, −0.159)**	**<0.001**	**<0.001**	**1.473 (1.127, 1.82)**	**<0.001**	**<0.001**	**1.409 (1.064, 1.754)**	**<0.001**	**<0.001**
Late-preterm (34–36W)	**−0.275 (−0.332, −0.219)**	**<0.001**	**<0.001**	**−0.201 (−0.258, −0.145)**	**<0.001**	**<0.001**	**0.878 (0.719, 1.037)**	**<0.001**	**<0.001**	**0.832 (0.673, 0.991)**	**<0.001**	**<0.001**
Early-term (37–38W)	**−0.091 (−0.129, −0.053)**	**<0.001**	**<0.001**	**−0.068 (−0.106, −0.031)**	**<0.001**	**<0.001**	**0.200 (0.094, 0.307)**	**<0.001**	**<0.001**	**0.233 (0.127, 0.339)**	**<0.001**	**<0.001**
Full-term (39–40W)	Reference			Reference			Reference			Reference		
Late-term (41–41W)	**0.072 (0.005, 0.138)**	**0.035**	**0.0035**	**0.067 (0.001, 0.133)**	**0.048**	**0.048**	0.075 (**−**0.112, 0.263)	0.430	0.430	0.005 (**−**0.182, 0.191)	0.959	0.959
Post-term (>41W)	**−0.166 (−0.240, −0.092)**	**<0.001**	**<0.001**	**−0.110 (−0.184, −0.036)**	**0.003**	**0.0036**	**0.901 (0.693, 1.109)**	**<0.001**	**<0.001**	**0.831 (0.624, 1.039)**	**<0.001**	**<0.001**
**3 years old**												
Very-preterm(<31W)	**−0.54 (−0.735, −0.345)**	**<0.001**	**<0.001**	**−0.473 (−0.667, −0.279)**	**<0.001**	**<0.001**	**2.240 (1.704, 2.776)**	**<0.001**	**<0.001**	**2.154 (1.620, 2.688)**	**<0.001**	**<0.001**
Moderate-preterm(32–33W)	**−0.448 (−0.675, −0.221)**	**<0.001**	**<0.001**	**−0.397 (−0.623, −0.171)**	**0.001**	**0.002**	**1.836 (1.211, 2.46)**	**<0.001**	**<0.001**	**1.810 (1.188, 2.433)**	**<0.001**	**<0.001**
Late-preterm (34–36W)	**−0.256 (−0.357, −0.155)**	**<0.001**	**<0.001**	**−0.191 (−0.291, −0.09)**	**<0.001**	**<0.001**	**0.588 (0.31, 0.865)**	**<0.001**	**<0.001**	**0.546 (0.268, 0.823)**	**<0.001**	**<0.001**
Early-term (37–38W)	**−0.077 (−0.141, −0.014)**	**0.017**	**0.0204**	**−**0.059 (**−**0.123, 0.004)	0.068	0.081	0.180 (0.005, 0.356)	**0.044**	0.052	**0.231 (0.056, 0.406)**	**0.01**	**0.012**
Full-term (39–40W)	Reference			Reference			Reference			Reference		
Late-term (41–41W)	**−**0.028 (**−**0.139, 0.082)	0.617	0.617	**−**0.032 (**−**0.142, 0.078)	0.571	0.571	**−**0.074 (**−**0.379, 0.231)	0.634	0.634	**−**0.163 (**−**0.467, 0.141)	0.293	0.293
Post-term (>41W)	**−0.215 (−0.348, −0.083)**	**0.001**	**0.0015**	**−0.166 (−0.298, −0.034)**	**0.014**	**0.021**	**0.927 (0.562, 1.292)**	**<0.001**	**<0.001**	**0.845 (0.481, 1.209)**	**<0.001**	**<0.001**
**4 years old**												
Very-preterm(<31W)	**−0.315 (−0.491, −0.14)**	**<0.001**	**<0.001**	**−0.248 (−0.423, −0.073)**	**0.005**	**0.010**	**1.771 (1.279, 2.263)**	**<0.001**	**<0.001**	**1.648 (1.157, 2.139)**	**<0.001**	**<0.001**
Moderate-preterm(32–33W)	**−0.237 (−0.43, −0.043)**	**0.016**	**0.024**	**−**0.161 (**−**0.353, 0.032)	0.102	0.122	**1.472 (0.93, 2.014)**	**<0.001**	**<0.001**	**1.374 (0.833, 1.914)**	**<0.001**	**<0.001**
Late-preterm (34–36W)	**−0.236 (−0.328, −0.145)**	**<0.001**	**<0.001**	**−0.166 (−0.258, −0.074)**	**<0.001**	**<0.001**	**1.018 (0.762, 1.275)**	**<0.001**	**<0.001**	**0.954 (0.697, 1.211)**	**<0.001**	**<0.001**
Early-term (37–38W)	**−0.113 (−0.174, −0.051)**	**<0.001**	**<0.001**	**−0.089 (−0.151, −0.028)**	**0.004**	**0.010**	**0.255 (0.083, 0.427)**	**0.0036**	**0.0043**	**0.283 (0.111, 0.455)**	**0.001**	**0.0012**
Full-term (39–40W)	Reference			Reference			Reference			Reference		
Late-term (41–41W)	**0.118 (0.012, 0.224)**	**0.029**	**0.0348**	0.109 (0.004, 0.215)	**0.042**	0.063	**0.308 (0.011, 0.605)**	**0.042**	**0.042**	0.262 (**−**0.034, 0.557)	0.083	0.083
Post-term (>41W)	**−0.126 (−0.244, −0.009)**	**0.035**	**0.035**	**−**0.077 (**−**0.194, 0.04)	0.198	0.198	**1.022 (0.692, 1.352)**	**<0.001**	**<0.001**	**0.900 (0.571, 1.229)**	**<0.001**	**<0.001**
**5 years old**												
Very-preterm(<31W)	**−0.254 (−0.462, −0.047)**	**0.016**	**0.032**	**−**0.196 (**−**0.403, 0.011)	0.063	0.094	**1.823 (1.227, 2.419)**	**<0.001**	**<0.001**	**1.731 (1.137, 2.326)**	**<0.001**	**<0.001**
Moderate-preterm(32–33W)	**−0.37 (−0.597, −0.144)**	**0.001**	**0.003**	**−0.329 (−0.555, −0.104)**	**0.004**	**0.012**	**1.221 (0.57, 1.872)**	**<0.001**	**<0.001**	**1.112 (0.463, 1.761)**	**<0.001**	**<0.001**
Late-preterm (34–36W)	**−0.31 (−0.414, −0.207)**	**<0.001**	**<0.001**	**−0.249 (−0.353, −0.146)**	**<0.001**	**<0.001**	**1.046 (0.749, 1.343)**	**<0.001**	**<0.001**	**0.953 (0.655, 1.251)**	**0.001**	**0.0015**
Early-term (37–38W)	**−**0.074 (**−**0.147, **−**0.001)	**0.046**	0.0552	**−**0.05 (**−**0.123, 0.023)	0.182	0.182	0.162 (**−**0.048, 0.372)	0.130	0.156	0.176 (**−**0.034, 0.386)	0.101	0.120
Full-term (39–40W)	Reference			Reference			Reference			Reference		
Late-term (41–41W)	0.124 (**−**0.011, 0.259)	0.071	0.071	0.129 (**−**0.005, 0.264)	0.056	0.094	**−**0.112 (**−**0.499, 0.275)	0.571	0.571	**−**0.175 (**−**0.561, 0.211)	0.375	0.375
Post-term (>41W)	**−**0.145 (**−**0.282, **−**0.007)	**0.039**	0.0552	**−**0.091 (**−**0.228, 0.046)	0.179	0.182	**0.78 (0.385, 1.175)**	**<0.001**	**<0.001**	**0.724 (0.330, 1.118)**	**<0.001**	**<0.001**

Daily sleep hours=5/7*sleep hours on weekdays +2/7*sleep hours at the weekends

*CSHQ* Children’s Sleep Habit Questionnaire, *CI* confidence interval

^a^Adjusted for age, gender, eyesight, co-sleep, maternal characteristics and family characteristics

p* value corrected after multiple testing

^b^Statistically significant results (*p* < 0.05) are in bold

The associations between very-preterm, moderate-preterm, late-preterm and post-term categories with the CSHQ subscale scores were found in six subscales but not bedtime resistance or sleep onset delay (Table 5).

**Table 5:** The age-specific association between gestational age with subscale score of CSHQ in preschoolers of different age (*n*=114,311)

Gestational age	Bedtime resistance						Sleep onset delay					
	Crude *β* (95% CI)	*p*	p*	Adjusted *β* ^a^ (95% CI)	*p*	p*	Crude *β* (95% CI)	*p*	p*	Adjusted *β* ^a^ (95% CI)	*p*	p*
Total												
Very-preterm(<31W)	0.056 (−0.036, 0.149)	0.234	0.2808	0.116 (0.025, 0.207)	0.012	0.036	0.057 (0.028, 0.085)	<0.001	<0.001	0.049 (0.021, 0.078)	0.001	0.006
Moderate-preterm(32–33W)	0.08 (−0.023, 0.183)	0.129	0.1935	0.149 (0.047, 0.25)	0.004	0.024	0.054 (0.022, 0.086)	0.001	0.003	0.047 (0.016, 0.079)	0.003	0.009
Late-preterm (34–36W)	−0.037 (−0.084, 0.011)	0.128	0.1935	0.048 (0.002, 0.095)	0.043	0.082	0.021 (0.007, 0.036)	0.005	0.010	0.016 (0.001, 0.031)	0.032	0.064
Early-term (37–38W)	−0.046 (−0.078, −0.014)	0.004	0.012	−0.008 (−0.04, 0.023)	0.603	0.603	−0.001 (−0.011, 0.008)	0.772	0.772	−0.002 (−0.011, 0.008)	0.745	0.745
Full-term (39–40W)	Reference			Reference			Reference			Reference		
Late-term (41–41W)	0.088 (0.032, 0.144)	0.002	0.012	0.054 (−0.001, 0.109)	0.055	0.082	0.017 (0, 0.034)	0.057	0.085	0.012 (−0.005, 0.03)	0.160	0.240
Post-term (>41W)	−0.003 (−0.065, 0.059)	0.921	0.921	0.052 (−0.009, 0.113)	0.096	0.115	0.013 (−0.006, 0.032)	0.183	0.219	0.01 (−0.009, 0.029)	0.323	0.387
3 years old												
Very-preterm(<31W)	0.095 (−0.066, 0.256)	0.249	0.394	0.149 (−0.010, 0.308)	0.067	0.402	0.095 (0.043, 0.147)	<0.001	<0.001	0.090 (0.038, 0.142)	0.001	0.006
Moderate-preterm(32–33W)	0.065 (−0.123, 0.252)	0,498	0.597	0.125 (−0.060, 0.310)	0.187	0.561	0.076 (0.016, 0.136)	0.014	0.042	0.069 (0.009, 0.129)	0.025	0.075
Late-preterm (34–36W)	−0.107 (−0.19, −0.024)	0.012	0.072	−0.04 (−0.123, 0.042)	0.339	0.591	0.001 (−0.026, 0.027)	0.961	0.961	−0.004 (−0.031, 0.022)	0.743	0.743
Early-term (37–38W)	−0.053 (−0.105, 0.0001)	0.05	0.072	−0.018 (−0.07, 0.034)	0.493	0.591	−0.009 (−0.026, 0.008)	0.285	0.496	−0.008 (−0.025, 0.009)	0.382	0.573
Full-term (39–40W)	Reference			Reference			Reference			Reference		
Late-term (41–41W)	0.052 (−0.039, 0.144)	0.263	0.394	0.024 (−0.066, 0.115)	0.597	0.597	0.015 (−0.015, 0.044)	0.331	0.496	0.009 (−0.021, 0.038)	0.570	0.684
Post-term (>41W)	0.015 (−0.095, 0.124)	0.795	0.795	0.046 (−0.063, 0.154)	0.408	0.591	−0.011 (−0.046, 0.025)	0.551	0.661	−0.016 (−0.051, 0.019)	0.380	0.573
4 years old												
Very-preterm(<31W)	0.069 (−0.076, 0.215)	0.351	0.526	0.111 (−0.033, 0.255)	0.131	0.196	0.011 (−0.034, 0.056)	0.637	0.637	0.001 (−0.044, 0.046)	0.964	0.964
Moderate-preterm(32–33W)	0.13 (−0.03, 0.291)	0.112	0.234	0.183 (0.024, 0.342)	0.024	0.134	0.061 (0.011, 0.111)*	0.017	0.102	0.050 (0.0001, 0.1)	0.049	0.201
Late-preterm (34–36W)	0.007 (−0.069, 0.083)	0.855	0.904	0.07 (−0.005, 0.146)	0.067	0.134	0.008 (−0.016, 0.032)	0.508	0.609	0.001 (−0.023, 0.024)	0.948	0.964
Early-term (37–38W)	−0.041 (−0.092, 0.01)	0.117	0.234	−0.006 (−0.056, 0.045)	0.830	0.830	0.008 (−0.007, 0.024)	0.301	0.451	0.008 (−0.008, 0.024)	0.336	0.504
Full-term (39–40W)	Reference			Reference			Reference			Reference		
Late-term (41–41W)	0.111 (0.023, 0.199)*	0.014	0.084	0.081 (−0.006, 0.168)	0.067	0.134	0.028 (0.001, 0.056)	0.043	0.112	0.025 (−0.002, 0.053)	0.067	0.201
Post-term (>41W)	0.006 (−0.092, 0.104)	0.904	0.094	0.029 (−0.068, 0.125)	0.561	0.672	0.03 (−0.001, 0.06)	0.056	0.112	0.023 (−0.007, 0.054)	0.133	0.266
5 years old												
Very-preterm(<31W)	0.06 (−0.115, 0.235)	0.500	0.500	0.086 (−0.088, 0.259)	0.333	0.424	0.085 (0.033, 0.137)*	0.001	0.003	0.074 (0.022, 0.126)	0.005	0.015
Moderate-preterm(32–33W)	0.126 (−0.065, 0.317)	0.197	0.487	0.125 (−0.064, 0.314)	0.196	0.392	0.038 (−0.019, 0.095)	0.191	0.286	0.028 (−0.029, 0.085)	0.332	0.505
Late-preterm (34–36W)	0.071 (−0.016, 0.158)	0.112	0.487	0.102 (0.015, 0.189)	0.021	0.126	0.067 (0.041, 0.093)*	<0.001	<0.001	0.054 (0.028, 0.08)	<0.001	<0.001
Early-term (37–38W)	−0.031 (−0.093, 0.031)	0.325	0.487	−0.003 (−0.064, 0.059)	0.934	0.934	−0.003 (−0.022, 0.015)	0.710	0.852	−0.006 (−0.025, 0.012)	0.490	0.588
Full-term (39–40W)	Reference			Reference			Reference			Reference		
Late-term (41–41W)	0.059 (−0.055, 0.173)	0.311	0487	0.053 (−0.059, 0.166)	0.354	0.424	−0.003 (−0.037, 0.031)	0.860	0.860	−0.006 (−0.04, 0.027)	0.709	0.709
Post-term (>41W)	0.049 (−0.067, 0.165)	0.411	0.493	0.084 (−0.031, 0.199)	0.151	0.392	0.025 (−0.01, 0.059)	0.162	0.286	0.017 (−0.018, 0.051)	0.337	0.505
Gestational age	Night wakings						Parasomnia					
	Crude *β* (95% CI)	*p*	p*	Adjusted *β* ^a^ (95% CI)	*p*	p*	Crude *β* (95% CI)	*p*	p*	Adjusted *β* ^a^ (95% CI)	*p*	p*
Total												
Very-preterm(<31W)	0.237 (0.191, 0.282)	<0.001	<0.01	0.227 (0.181, 0.272)	<0.001	<0.01	0.526 (0.433, 0.619)	<0.001	<0.001	0.515 (0.422, 0.608)	<0.001	<0.001
Moderate-preterm(32–33W)	0.166 (0.116, 0.217)	<0.001	<0.001	0.163 (0.112, 0.214)	<0.001	<0.001	0.341 (0.237, 0.445)	<0.001	<0.001	0.337 (0.233, 0.441)	<0.001	<0.001
Late-preterm (34–36W)	0.122 (0.099, 0.146)	<0.001	<0.001	0.117 (0.093, 0.14)	<0.001	<0.001	0.241 (0.193, 0.288)	<0.001	<0.001	0.238 (0.19, 0.286)	<0.001	<0.001
Early-term (37–38W)	0.036 (0.02, 0.052)	<0.001	<0.001	0.037 (0.022, 0.053)	<0.001	<0.001	0.079 (0.047, 0.111)	<0.001	<0.001	0.085 (0.053, 0.117)	<0.001	<0.001
Full-term (39–40W)	Reference			Reference			Reference			Reference		
Late-term (41–41W)	−0.012 (−0.04, 0.015)	0.386	0.386	−0.018 (−0.046, 0.009)	0.190	0.190	−0.001 (−0.057, 0.056)	0.983	0.983	−0.011 (−0.067, 0.045)	0.694	0.694
Post-term (>41W)	0.089 (0.059, 0.12)	<0.001	<0.001	0.083 (0.052, 0.113)	<0.001	<0.001	0.228 (0.165, 0.29)	<0.001	<0.001	0.221 (0.158, 0.283)	<0.001	<0.001
3years old												
Very-preterm(<31W)	0.246 (0.163, 0.329)	<0.001	<0.01	0.24 (0.157, 0.323)	<0.001	<0.01	0.607 (0.445, 0.769)	<0.001	<0.01	0.599 (0.437, 0.76)	<0.001	<0.01
Moderate-preterm(32–33W)	0.21 (0.113, 0.307)	<0.001	<0.001	0.213 (0.117, 0.31)	<0.001	<0.001	0.459 (0.27, 0.648)	<0.001	<0.001	0.464 (0.275, 0.652)	<0.001	<0.001
Late-preterm (34–36W)	0.083 (0.04, 0.126)	<0.001	<0.001	0.082 (0.039, 0.126)	<0.001	<0.001	0.172 (0.088, 0.256)	<0.001	<0.001	0.174 (0.09, 0.258)	<0.001	<0.001
Early-term (37–38W)	0.024 (−0.003, 0.052)	0.078	0.0936	0.029 (0.002, 0.056)	0.035	0.042	0.07 (0.017, 0.123)	0.009	0.010	0.081 (0.028, 0.134)	0.003	0.0036
Full-term (39–40W)	Reference			Reference			Reference			Reference		
Late-term (41–41W)	0.001 (−0.047, 0.047)	0.995	0.995	−0.007 (−0.055, 0.04)	0.759	0.759	−0.003 (−0.096, 0.089)	0.941	0.941	−0.017 (−0.109, 0.075)	0.715	0.715
Post-term (>41W)	0.093 (0.036, 0.149)	<0.001	<0.001	0.089 (0.033, 0.146)	0.002	0.003	0.217 (0.107, 0.327)	<0.001	<0.001	0.212 (0.102, 0.322)	<0.001	<0.001
4 years old												
Very-preterm(<31W)	0.23 (0.158, 0.302)	<0.001	<0.01	0.213 (0.142, 0.285)	<0.001	<0.01	0.511 (0.363, 0.658)	<0.001	<0.01	0.493 (0.345, 0.64)	<0.001	<0.01
Moderate-preterm(32–33W)	0.157 (0.078, 0.236)	<0.001	<0.001	0.141 (0.062, 0.22)	<0.001	<0.001	0.29 (0.127, 0.452)	<0.001	<0.001	0.277 (0.115, 0.44)	0.001	0.0012
Late-preterm (34–36W)	0.159 (0.121, 0.196)	<0.001	<0.001	0.144 (0.106, 0.182)	<0.001	<0.001	0.283 (0.207, 0.36)	<0.001	<0.001	0.273 (0.195, 0.35)	<0.001	<0.001
Early-term (37–38W)	0.037 (0.012, 0.062)	0.004	0.0048	0.038 (0.013, 0.063)	0.003	0.0036	0.083 (0.032, 0.135)	0.002	0.0024	0.087 (0.035, 0.138)	0.001	0.012
Full-term (39–40W)	Reference			Reference			Reference			Reference		
Late-term (41–41W)	−0.006 (−0.049, 0.038)	0.801	0.801	−0.008 (−0.052, 0.035)	0.709	0.709	0.055 (−0.035, 0.144)	0.230	0.230	0.05 (−0.039, 0.139)	0.274	0.274
Post-term (>41W)	0.107 (0.058, 0.155)	<0.001	<0.001	0.09 (0.041, 0.138)	<0.001	<0.001	0.293 (0.194, 0.392)	<0.001	<0.001	0.274 (0.175, 0.373)	<0.001	<0.001
5 years old												
Very-preterm(<31W)	0.249 (0.165, 0.333)	<0.001	<0.01	0.233 (0.15, 0.317)	<0.001	<0.01	0.478 (0.301, 0.656)	<0.001	<0.01	0.461 (0.284, 0.639)	<0.001	<0.01
Moderate-preterm(32–33W)	0.161 (0.069, 0.252)	0.001	0.0015	0.15 (0.059, 0.242)	0.001	0.002	0.326 (0.132, 0.52)	0.001	<0.001	0.312 (0.118, 0.506)	0.002	0.004
Late-preterm (34–36W)	0.132 (0.09, 0.174)	<0.001	<0.001	0.116 (0.075, 0.158)	<0.001	<0.001	0.271 (0.182, 0.359)	0.001	0.002	0.253 (0.164, 0.343)	<0.001	<0.001
Early-term (37–38W)	0.05 (0.021, 0.08)	0.001	0.0015	0.047 (0.017, 0.076)	0.002	0.003	0.084 (0.021, 0.146)	0.009	0.0108	0.085 (0.022, 0.148)	0.008	0.009
Full-term (39–40W)	Reference			Reference			Reference			Reference		
Late-term (41–41W)	−0.045 (−0.099, 0.01)	0.107	0.107	−0.051 (−0.105, 0.003)	0.065	0.065	−0.086 (−0.202, 0.029)	0.143	0.143	−0.095 (−0.211, 0.02)	0.106	0.106
Post-term (>41W)	0.081 (0.026, 0.136)	0.004	0.0048	0.069 (0.014, 0.124)	0.014	0.016	0.175 (0.057, 0.293)	0.004	0.006	0.164 (0.046, 0.282)	0.006	0.009

Gestational age	Sleep duration										Sleep anxiety						
	Crude *β* (95% CI)		*p*		p*		Adjusted *β* ^a^ (95% CI)		*p*	p*	Crude *β* (95% CI)		*p*	p*	Adjusted *β* ^a^ (95% CI)	*p*	p*
Total																	
Very-preterm(<31W)	0.255 (0.193, 0.316)		<0.001		<0.01		0.194 (0.133, 0.256)		<0.001	<0.001	0.241 (0.175, 0.308)		<0.001	<0.01	0.180 (0.114, 0.246)	<0.001	<0.001
Moderate-preterm(32–33W)	0.235 (0.166, 0.304)		<0.001		<0.001		0.17 (0.102, 0.239)		<0.001	<0.001	0.225 (0.151, 0.299)		<0.001	<0.001	0.160 (0.087, 0.233)	<0.001	<0.001
Late-preterm (34–36W)	0.149 (0.118, 0.181)		<0.001		<0.001		0.078 (0.047, 0.11)		<0.001	<0.001	0.14 (0.106, 0.174)		<0.001	<0.001	0.069 (0.035, 0.103)	<0.001	<0.001
Early-term (37–38W)	0.036 (0.014, 0.057)		0.001		0.0012		0.012 (−0.009, 0.033)		0.255	0.306	0.035 (0.012, 0.057)		0.003	0.0036	0.011 (−0.011, 0.034)	0.324	0.388
Full-term (39–40W)	Reference						Reference				Reference						
Late-term (41–41W)	−0.002 (−0.039, 0.036)		0.937		0.937		0.006 (−0.031, 0.043)		0.740	0.740	−0.011 (−0.051, 0.029)		0.590	0.590	−0.003 (−0.042, 0.037)	0.890	0.890
Post-term (>41W)	0.128 (0.087, 0.17)		<0.001		<0.001		0.084 (0.043, 0.125)		<0.001	<0.001	0.121 (0.076, 0.165)		<0.001	<0.001	0.076 (0.032, 0.12)	0.001	0.0015
3 years old																	
Very-preterm(<31W)	0.338 (0.228, 0.449)		<0.001		<0.01		0.277 (0.168, 0.387)		<0.001	<0.01	0.341 (0.223, 0.459)		<0.001	<0.001	0.277 (0.16, 0.394)	<0.001	<0.01
Moderate-preterm(32–33W)	0.313 (0.184, 0.442)		<0.001		<0.001		0.253 (0.125, 0.381)		<0.001	<0.001	0.313 (0.178, 0.449)		<0.001	<0.001	0.255 (0.121, 0.39)	<0.001	<0.001
Late-preterm (34–36W)	0.159 (0.102, 0.216)		<0.001		<0.001		0.094 (0.037, 0.151)		0.001	0.002	0.139 (0.078, 0.199)		<0.001	<0.001	0.075 (0.015, 0.135)	0.015	0.030
Early-term (37–38W)	0.044 (0.008, 0.081)		0.016		0.019		0.024 (−0.012, 0.06)		0.186	0.223	0.041 (0.003, 0.079)		0.036	0.043	0.021 (−0.017, 0.059)	0.281	0.337
Full-term (39–40W)	Reference						Reference				Reference				Reference		
Late-term (41–41W)	0.001 (−0.063, 0.063)		0.989		0.989		0.006 (−0.056, 0.068)		0.851	0.851	−0.012 (−0.079, 0.054)		0.717	0.717	−0.006 (−0.072, 0.06)	0.854	0.854
Post-term (>41W)	0.116 (0.04, 0.191)		0.003		0.004		0.077 (0.003, 0.152)		0.043	0.064	0.094 (0.015, 0.172)		0.002	0.003	0.056 (−0.022, 0.135)	0.157	0.235
4 years old																	
Very-preterm(<31W)	0.199 (0.1, 0.297)		<0.001		<0.01		0.142 (0.044, 0.24)		0.005	0.010	0.192 (0.086, 0.297)		<0.001	<0.01	0.136 (0.031, 0.241)	0.011	0.024
Moderate-preterm(32–33W)	0.239 (0.131, 0.348)		<0.001		<0.001		0.176 (0.069, 0.284)		0.001	0.006	0.212 (0.096, 0.328)		<0.001	<0.001	0.149 (0.033, 0.264)	0.012	0.024
Late-preterm (34–36W)	0.136 (0.085, 0.187)		<0.001		<0.001		0.075 (0.023, 0.126)		0.004	0.010	0.13 (0.075, 0.185)		<0.001	<0.001	0.071 (0.016, 0.126)	0.011	0.024
Early-term (37–38W)	0.041 (0.006, 0.075)		0.020		0.0240		0.018 (−0.017, 0.052)		0.310	0.310	0.047 (0.01, 0.083)		0.013	0.015	0.023 (−0.013, 0.06)	0.213	0.255
Full-term (39–40W)	Reference						Reference				Reference				Reference		
Late-term (41–41W)	0.022 (−0.037, 0.082)		0.462		0.462		0.031 (−0.028, 0.09)		0.307	0.310	0.017 (−0.047, 0.08)		0.606	0.606	0.027 (−0.036, 0.09)	0.400	0.400
Post-term (>41W)	0.101 (0.035, 0.167)		0.003		0.004		0.065 (0, 0.131)		0.051	0.076	0.101 (0.03, 0.172)		0.005	0.007	0.064 (−0.007, 0.134)	0.076	0.114
5 years old																	
Very-preterm(<31W)	0.222 (0.108, 0.336)		<0.001		<0.001		0.178 (0.065, 0.291)		0.002	0.009	0.177 (0.055, 0.299)		0.005	0.010	0.137 (0.015, 0.258)	0.028	0.048
Moderate-preterm(32–33W)	0.127 (0.003, 0.251)		0.045		0.067		0.088 (−0.035, 0.211)		0.162	0.243	0.123 (−0.013, 0.258)		0.076	0.114	0.085 (−0.05, 0.219)	0.219	0.285
Late-preterm (34–36W)	0.125 (0.068, 0.181)		<0.001		<0.001		0.069 (0.013, 0.126)		0.016	0.032	0.119 (0.058, 0.181)		<0.001	<0.001	0.062 (0.001, 0.123)	0.048	0.096
Early-term (37–38W)	0.015 (−0.026, 0.055)		0.477		0.477		−0.008 (−0.048, 0.032)		0.693	0.693	0.007 (−0.036, 0.051)		0.746	0.746	−0.015 (−0.059, 0.028)	0.488	0.488
Full-term (39–40W)	Reference						Reference				Reference				Reference		
Late-term (41–41W)	−0.028 (−0.102, 0.046)		0.458		0.477		−0.033 (−0.106, 0.041)		0.385	0.462	−0.041 (−0.122, 0.039)		0.313	0.375	−0.048 (−0.128, 0.032)	0.238	0.285
Post-term (>41W)	0.148 (0.073, 0.224)		<0.001		<0.001		0.113 (0.038, 0.188)		0.003	0.009	0.148 (0.066, 0.23)		<0.001	<0.001	0.111 (0.029, 0.192)	0.008	0.048
Gestational age	Sleep disorder breathing										Daytime sleepiness						
	Crude *β* (95% CI)	*p*		p*		Adjusted *β* ^a^ (95% CI)		*p*		p*	Crude *β* (95% CI)	*p*		p*	Adjusted *β* ^a^ (95% CI)	*p*	p*
Total																	
Very-preterm(<31W)	0.221 (0.182, 0.26)	<0.001		<0.01		0.208 (0.169, 0.247)		<0.001		<0.01	0.424 (0.315, 0.533)	<0.001		<0.01	0.377 (0.268, 0.486)	<0.001	<0.01
Moderate-preterm(32–33W)	0.157 (0.113, 0.2)	<0.001		<0.001		0.143 (0.1, 0.187)		<0.001		<0.001	0.32 (0.198, 0.441)	<0.001		<0.001	0.279 (0.158, 0.401)	<0.001	<0.001
Late-preterm (34–36W)	0.118 (0.098, 0.138)	<0.001		<0.001		0.105 (0.085, 0.125)		<0.001		<0.001	0.196 (0.14, 0.252)	<0.001		<0.001	0.161 (0.105, 0.217)	<0.001	<0.001
Early-term (37–38W)	0.036 (0.022, 0.049)	<0.001		<0.001		0.034 (0.021, 0.047)		<0.001		<0.001	0.04 (0.002, 0.077)*	0.038		0.0456	0.049 (0.012, 0.086)	0.010	0.012
Full-term (39–40W)	Reference					Reference					Reference				Reference		
Late-term (41–41W)	−0.022 (−0.046, 0.001)	0.064		0.064		−0.022 (−0.046, 0.002)		0.067		0.067	0.013 (−0.053, 0.079)	0.699		0.699	−0.007 (−0.072, 0.059)	0.843	0.843
Post-term (>41W)	0.097 (0.071, 0.123)	<0.001		<0.001		0.085 (0.059, 0.111)		<0.001		<0.001	0.29 (0.217, 0.364)	<0.001		<0.001	0.242 (0.169, 0.314)	<0.001	<0.001
3years old																	
Very-preterm(<31W)	0.275 (0.21, 0.339)	<0.001		<0.01		0.267 (0.202, 0.332)		<0.001		<0.01	0.42 (0.223, 0.617)	<0.001		<0.01	0.369 (0.174, 0.565)	<0.001	<0.01
Moderate-preterm(32–33W)	0.123 (0.048, 0.199)	0.001		0.0015		0.117 (0.041, 0.192)		0.002		0.003	0.401 (0.171, 0.63)	0.001		0.0015	0.377 (0.149, 0.605)	0.001	0.0015
Late-preterm (34–36W)	0.062 (0.028, 0.095)	<0.001		<0.001		0.056 (0.022, 0.09)		0.001		0.002	0.168 (0.067, 0.27)	0.001		0.0015	0.133 (0.031, 0.234)	0.010	0.0015
Early-term (37–38W)	0.028 (0.007, 0.049)	0.010		0.0012		0.029 (0.008, 0.05)		0.008		0.0096	0.038 (−0.026, 0.102)	0.246		0.246	0.052 (−0.012, 0.116)	0.112	0.112
Full-term (39–40W)	Reference					Reference					Reference				Reference		
Late-term (41–41W)	−0.026 (−0.063, 0.011)	0.168		0.168		−0.029 (−0.065, 0.008)		0.129		0.129	−0.099 (−0.211, 0.013)	0.084		0.1008	−0.131 (−0.242, −0.02)	0.021	0.025
Post-term (>41W)	0.088 (0.044, 0.133)	<0.001		<0.001		0.081 (0.037, 0.125)		0.001		0.002	0.335 (0.201, 0.468)	<0.001		<0.001	0.283 (0.15, 0.416)	<0.001	<0.001
4 years old																	
Very-preterm(<31W)	0.179 (0.117, 0.241)	<0.001		<0.01		0.165 (0.103, 0.228)		<0.001		<0.01	0.444 (0.27, 0.617)	<0.001		<0.01	0.395 (0.222, 0.568)	<0.001	<0.01
Moderate-preterm(32–33W)	0.16 (0.092, 0.229)	<0.001		<0.001		0.148 (0.08, 0.216)		<0.001		<0.001	0.33 (0.139, 0.521)	0.001		0.0015	0.291 (0.101, 0.481)	0.003	0.0036
Late-preterm (34–36W)	0.139 (0.107, 0.171)	<0.001		<0.001		0.127 (0.095, 0.16)		<0.001		<0.001	0.2 (0.109, 0.291)	0.001		0.0015	0.175 (0.084, 0.265)	<0.001	<0.001
Early-term (37–38W)	0.03 (0.008, 0.052)	0.007		0.0084		0.028 (0.006, 0.05)		0.011		0.0132	0.084 (0.023, 0.144)	0.007		0.0084	0.093 (0.033, 0.154)	0.003	0.0036
Full-term (39–40W)	Reference					Reference					Reference				Reference		
Late-term (41–41W)	−0.009 (−0.047, 0.028)	0.630		0.630		−0.008 (−0.046, 0.029)		0.658		0.658	0.099 (−0.006, 0.203)	0.065		0.065	0.087 (−0.017, 0.191)	0.102	0.102
Post-term (>41W)	0.115 (0.073, 0.157)	<0.001		<0.001		0.104 (0.062, 0.145)		<0.001		<0.001	0.313 (0.197, 0.43)	<0.001		<0.001	0.262 (0.146, 0.378)	<0.001	<0.001
5 years old																	
Very-preterm(<31W)	0.217 (0.14, 0.295)	<0.001		<0.01		0.205 (0.128, 0.282)		<0.001		<0.01	0.377 (0.177, 0.576)	<0.001		<0.01	0.36 (0.161, 0.558)	0.001	0.003
Moderate-preterm(32–33W)	0.174 (0.09, 0.258)	<0.001		<0.001		0.163 (0.079, 0.247)		<0.001		<0.001	0.194 (−0.023, 0.412)	0.080		0.120	0.172 (−0.045, 0.389)	0.120	0.180
Late-preterm (34–36W)	0.136 (0.098, 0.175)	<0.001		<0.001		0.124 (0.085, 0.162)		<0.001		<0.001	0.184 (0.085, 0.284)	<0.001		<0.001	0.174 (0.074, 0.273)	0.001	0.003
Early-term (37–38W)	0.052 (0.024, 0.079)	<0.001		<0.001		0.049 (0.021, 0.076)		<0.001		<0.015	−0.019 (−0.089, 0.051)	0.599		0.599	−0.006 (−0.076, 0.065)	0.877	0.877
Full-term (39–40W)	Reference					Reference					Reference				Reference		
Late-term (41–41W)	−0.034 (−0.084, 0.016)	0.184		0.184		−0.035 (−0.085, 0.015)		0.166		0.166	0.042 (−0.088, 0.171)	0.528		0.599	0.011 (−0.118, 0.14)	0.868	0.877
Post-term (>41W)	0.075 (0.023, 0.126)	0.004		0.0048		0.067 (0.016, 0.118)		0.010		0.012	0.188 (0.056, 0.321)#	0.005		0.010	0.169 (0.037, 0.300)	0.012	0.024

*CSHQ* Children’s Sleep Habit Questionnaire, *CI* confidence interval, *OR* odds ratio

^a^Adjusted for age, gender, eyesight, co-sleep, maternal characteristics, and family characteristics

p* value corrected after multiple testing

^b^Statistically significant results (*p* < 0.05) are in bold

Gestational age predicted pediatric sleep disorder (i.e. CHSQ>41; adjusted odds ratio (AOR) = 1.287 95% CI (1.157, 1.433), 1.249 95% CI (1.110, 1.405), 1.111 95%CI (1.052, 1·174), and 1.139 95%CI (1.061, 1.222), for the very-preterm, moderate-preterm, late-preterm, and post-term birth, respectively, each *p<*0.001, *p correction* <0.001, Table 3), after controlling for all the confounders and competing exposures. The associations were also evident in different age groups, mainly in the preterm and post-term categories (Table 3).

**Fig. 2:**
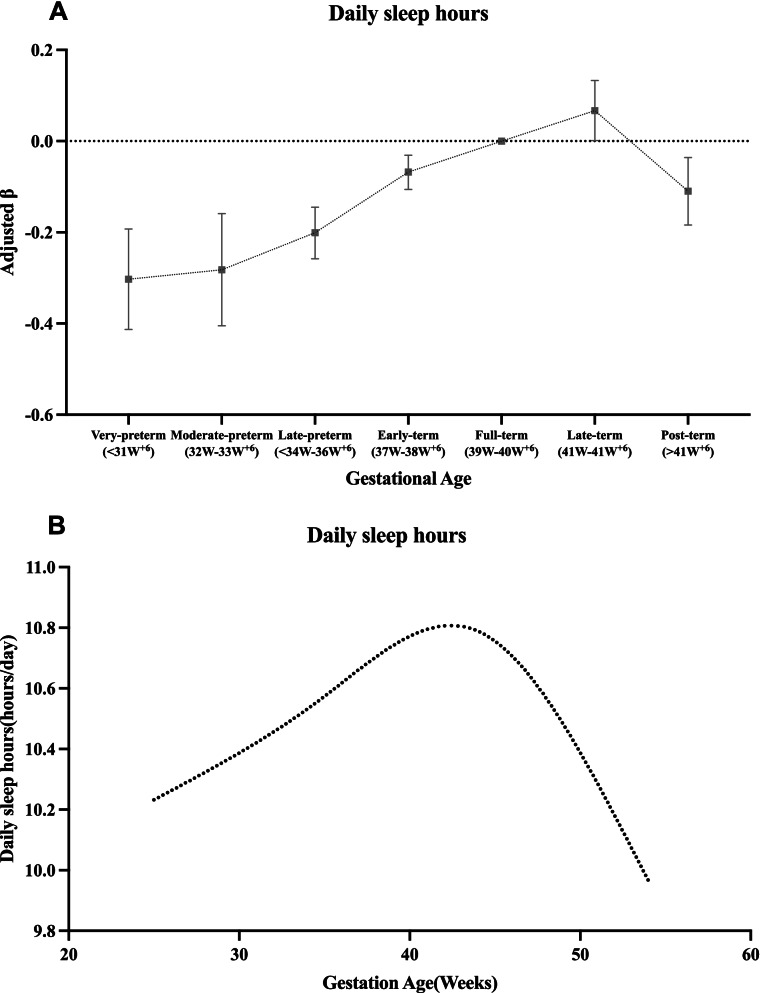
Associations of gestational age with daily sleep hours. Legend: Associations of gestational age with daily sleep hours: **A** Compared with full-term birth, preschool children born very-preterm, moderate-preterm, late-preterm, early-term and post-term were associated with lower daily sleep hours, all *p* < 0.01. **B** A fit spline described an inverse U-shape relationship of gestational age (weeks) with daily sleep hours (hours/day)

**Fig. 3:**
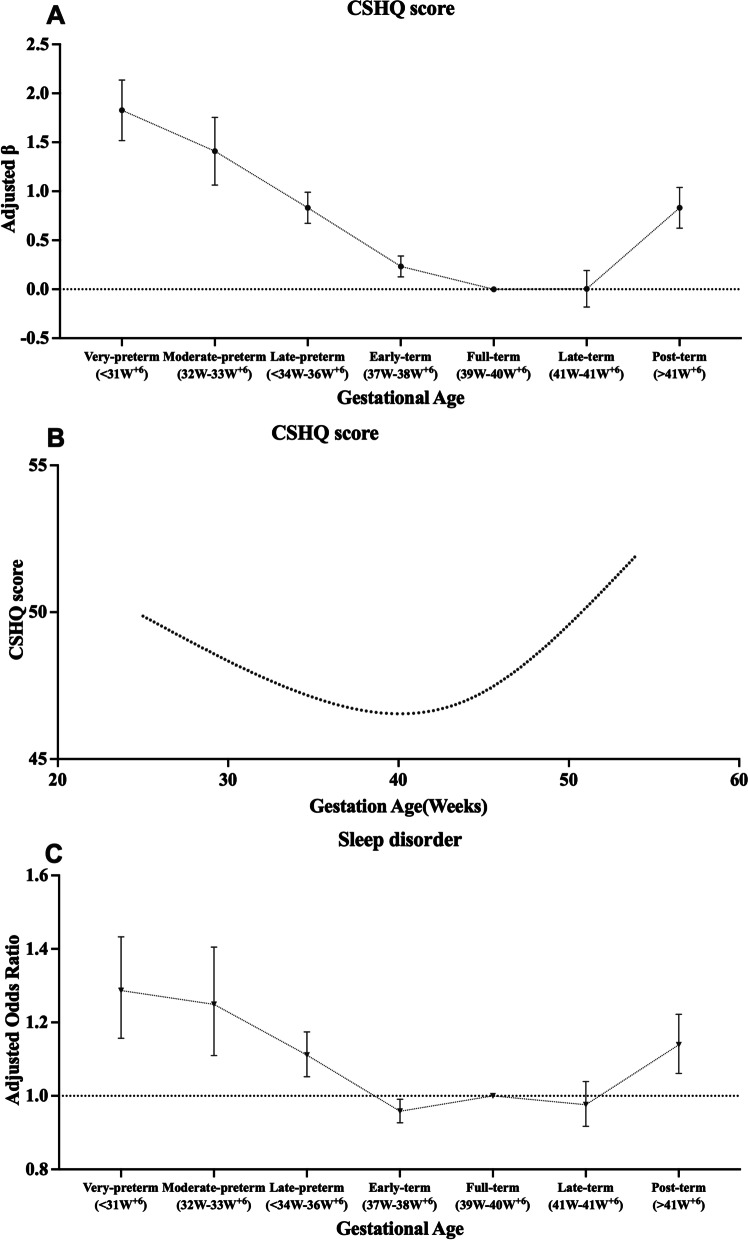
Associations of gestational age with CSHQ score and sleep disorder. Legend: Associations of gestational age with CSHQ scores and sleep disorder. **A** Compared with full-term birth, preschool children born very-preterm, moderate-preterm, late-preterm, early-term and post-term were associated with higher CSHQ scores, all *p* < 0.001. **B** A fit spline described a U-shape relationship of gestational age (weeks) with CSHQ scores. **C** Compared with full-term birth, preschool children born very-preterm, moderate-preterm, late-preterm and post-term were associated with a higher prevalence of pediatric sleep disorder, all *p* < 0.001

Based on these results in linear and logistic regression models, the associations between gestational ages and childhood daily sleep hours and childhood sleep disorders have been established, especially in preterm (including very-preterm, moderate-preterm and late-preterm) and post-term when compared with completely full-term birth (Figs. 2 and 3).

## Discussion

The current population-based prospective cohort study was the first to investigate the association between gestational age across the full range of sleep outcomes in preschoolers. Our study demonstrated that 3–5-year-old children born very-preterm (<31 weeks), moderate-preterm (32–33 weeks), late-preterm (34–36 weeks), early-term (37–38 weeks) and post-term (>41 weeks) were more likely to have sleep problems including having shorter daily sleep hours and a higher odds of sleep disorders, compared to their full-term-born peers, as reported by their parents. Moreover, preterm and post-term children have different sleep profiles as suggested by the different subscales of the CSHQ.

In the present study, a prevalence of sleep disorders was reported with a nationally representative sample in China. Sleep disorder defined with a CSHQ score higher than 41 was found to be prevalent in up to 81.27% of the very-preterm group, 80.67% of the moderate-preterm group, 78.62% of the late-preterm group, 76.58% of the early-term group, 76.02% of the full-term group and 79.17% of the post-term group. The remarkably high incidence of CHSQ-indicated sleep disorders is consistent with previous studies which also reported a very high prevalence of sleep disorders measured by the CSHQ in Chinese [41–44] and Japanese preschoolers [41], compared to a prevalence of sleep disorders reported in other populations 20–30% in the western population [2, 45]. Our results suggest the cultural features in sleep behaviours of children, and a local standard is required when using the CSHQ to define sleep disorders in children in East Asian countries.

In the present study, we found that sleep disorder as defined by CSHQ scores was significantly higher in all preterm groups compared with the full-term group. Our findings were consistent with previous work which also found that preterm children were more likely to have adverse sleep outcomes compared with full-term children even beyond infancy. The association between preterm birth and sleep problems in children beyond infancy have been reported by studies in both preschool ages [46–48] and school ages [10], and our results further suggest that preterm-born preschoolers, especially very-preterm (<31 weeks), were more likely to have sleep disorder consistently across our age range from 3 to 5 years old. Moreover, when we compared individual sleep subscales in the CSHQ, including Bedtime Resistance, Sleep Onset Delay, Sleep Duration, Sleep Anxiety, Night Wakings, Parasomnias, Sleep Disordered Breathing and Daytime Sleepiness, a significant difference between the very-preterm/moderate-preterm and full-term groups was found in all subscales. The preterm groups also showed significantly shorter daily sleep hours compared to full-term children. These results suggest that preterm children may have a global sleep problem. The results are different from a previous study that found preterm preschoolers had higher total scores in the CSHQ but not on any of the subscales [47], which may be associated with their small sample sizes (137 preterms vs. 145 full-term children aged 4–6 years old). As demonstrated by previous studies, circadian rhythms of the foetus are developing in the second trimester and mature in the third trimester [49, 50]. The cycles of foetal activity were reported to show a significant increase in cycle length with advancing gestation [51]. The last few weeks of gestation (37–41 weeks) could therefore be considered as the critical period for sleep patterns as circadian rhythms are established. On the other hand, the shorter daily sleep duration and more sleep problems in bedtime resistance and sleep onset delay of preterm children may also reflect other factors associated with preterm birth. A growing body of evidence indicates that unmodulated parental care and noncircadian environmental conditions may be detrimental to the establishment of circadian rhythms [52–55]. More behaviour problems and more social segregation have also been observed in children born preterm compared to full-term [56, 57]. As a consequence, these factors may also contribute to the shorter sleep duration and sleep problems of preterm children. Extrinsic and behavioural aspects of the sleep problems of preterm children might be worth investigating in future research.

The current study also shows that some sleep outcomes of early-term children (37–38 weeks) were more likely to be affected compared with those of full-term children. Not only preterm birth but also early-term birth can cause disruption at specific periods during the development of neural connections for specific brain areas which can affect sleep patterns [58]. A systematic review has suggested that early-term infants had poorer outcomes in school performance, neurodevelopment, behaviour and emotional status and long-term social outcomes [58]. Sleep disturbance might be associated with a loss of active cortical and cerebellar development between 34 and 40 weeks of gestation [59]. A cohort study also found that early-term deliveries were associated with a higher rate of pediatric obstructive sleep apnea (OSA), which decreases gradually as gestational age advances [60]. The sleep problems of early-term-born children may be mild and the differences are only revealed with large samples, as in the current study. In the present study, the altered sleep outcomes in early-term-born children faded in the 5-year age group. The results further suggested that the mild sleep problems observed in 3- and 4-year-old early-term-born children can be moderated by biological maturation or relevant social factors as children mature. To our knowledge, our study is the first to report sleep problems in early-term birth, which suggests that children born early-term should also be monitored more carefully due to the higher odds of experiencing sleep problems.

One important finding of the current study is that an association between post-term birth (>41 weeks) and altered sleep outcomes were observed. Previous studies have suggested that post-term birth may be associated with a range of adverse neurological, developmental, behavioural and emotional outcomes in early childhood [14, 19, 61]. Mechanisms concerning post-term birth with a higher risk of sleep disorder and shorter daily sleep hours might involve placental deterioration or insufficiency causing foetal hypoxia or nutritional deficiencies, which in turn could result in injury to the foetal brain [62]. Meconium aspiration, which is more common in post-term birth, may result in neonatal asphyxia thereby increasing the risk for brain injury and later neurodevelopmental problems [62, 63]. Lower melatonin concentration in post-term children might also contribute to a higher risk of sleep problems in post-term-born children [64].

Moreover, it should also be noted that the post-term children had different sleep profiles from preterm children as shown by subscales of the CSHQ. Preterm children showed more difficulties getting to bed (i.e. Bedtime Resistance) and longer periods of awake before sleep (i.e. Sleep Onset Delay) compared to post-term children. There were similar rates of co-sleeping in our preterm and post-term groups, and differences in these sleep behaviour problems between children born preterm and post-term might reflect other parental styles of managing bedtime routines. For example, increased parental concerns with preterm-born children could result in earlier bedtimes [65]. Previous studies also found that increased time was spent in bed in preterm children, irrespective of sleep duration [7], thus arbitrarily reducing sleep efficiency. These findings suggested that parental concern related to preterm does not automatically lead to longer sleep durations, but may lead to a longer sleep onset delay of preterm children. Similarly, the bedtime resistance of preterm children may also be associated with the altered parent-child relationship and the parenting style [66]. Previous research has shown that children with difficulties falling asleep/bedtime resistance are more likely to have parents with a higher level of parental stress [67]. Extinction for bedtime resistance involves requiring children to go to bed and stay in bed and minimizing parental attention thereafter. However, if parents have increased concerns about preterm children, this can lead to the development of substantial bedtime resistance behaviours [68]. On the other hand, parents of post-term-born children may overlook the long-term effect of the prolonged gestation of their child [69], which protects these children from the possible influences of social factors that could cause the delay in sleep onset times and bedtime resistance. Moreover, the shorter daily sleep duration of post-term children compared to full-term births was only observed in the 3- and 4-year-old group, but not in the 5-year-old group, which also suggested the sleep duration of post-term children can be improved by biological maturation or relevant social factors such as a more structured daily sleep routine in nursery. These environmental and behavioural aspects of the sleep problems of preterm and post-term children will need to be further examined in future research.

## Strengths and limitations

The main strength of our study was that we included and controlled for a wide range of possible confounding variables including prenatal, antenatal, and postnatal characteristics, and child and family characteristics. Another strength was that we used a nationwide large population sample, and we examined the gestational ages across the full range from preterm to post-term. Our study also had several limitations. First, we did not include all the covariates when analysing the relationship of gestational age with sleep outcomes. For example, some covariates such as maternal sleep of the mothers during pregnancy were not included in the current study but have been shown to affect childhood sleep [30]. Second, the reliance on parent-report information may raise the possibility of a differential misclassification. For example, parents of preterm children, especially very-preterm, may exaggerate the sleep problems of their preterm-born children. Thus large-scale studies using objective measures of sleep such as polysomnography PSG or actigraphy instead of parent reports would add substantially to the theoretical and practical utility of future findings. Fourthly, multiple testing exists in our analysis which may increase the probability of false positives. However, we used the Benjamini-Hochberg false discovery rate method for correction. Finally, it should be noted that some of the reported effect sizes were relatively small. However, the results still have clinical and public health relevance as preterm, early-term and post-term birth can affect a large segment of the population, and the results of the current study can also provide evidence for determining a composite risk.

## Conclusions

This cohort study demonstrated that every degree of preterm, early-term and post-term birth negatively affected sleep outcomes, including an increased tendency of parent-reported pediatric sleep disorders and shorter daily sleep hours compared to full-term children. Preterm and post-term births also showed different sleep profiles with preterm children having more sleep behaviour problems. The findings address a much-needed gap in the literature to date and provide important new evidence to support the associations between gestational age and childhood sleep outcomes. The findings suggest that children born preterm, early-term and post-term should be monitored more carefully concerning their sleep health. The potential biological mechanisms between gestational age and childhood sleep should be further studied.

## Supplementary Information

**Additional file 1:** **Figure S1.** A directed acyclic graph (DAG) describing the relationship between gestational age with daily sleep hours and CSHQ score. Green lines represent paths associated with variables on the causal pathway and were not included in adjusted models.

## Data Availability

The datasets analysed in the current study are available from the corresponding authors on reasonable request.

## Abbreviations

ANOVA: Analysis of variance
AOR: Adjusted odds ratio
ASD: Autism spectrum disorder
BMI: Body mass index
CI: Confidence interval
CNCMD: The Chinese National Cohort of Motor Development
CSHQ: The Children’s Sleep Habits Questionnaire
DAG: Directed acyclic graph
ICD 10: The International Classification of Diseases, Revision 10
NICU: Newborn intensive care unit
OSA: Obstructive sleep apnea
SES: Socioeconomic status
SPSS: Statistical Package for the Social Sciences
