# Supplementary material for: Associations between gestational age and childhood sleep: a national retrospective cohort study
Source: BMC Med. 2022 Aug 8;20:253. doi: 10.1186/s12916-022-02443-9 (PMC9358861; doi:10.1186/s12916-022-02443-9)
Supplement: Supplementary file 1 — Additional file 1: Figure S1. A directed acyclic graph (DAG) describing the relationship between gestational age with daily sleep hours and CSHQ score. Green lines represent paths associated with variables on the causal pathway and were not included in adjusted models. [file 12916_2022_2443_MOESM1_ESM.docx]

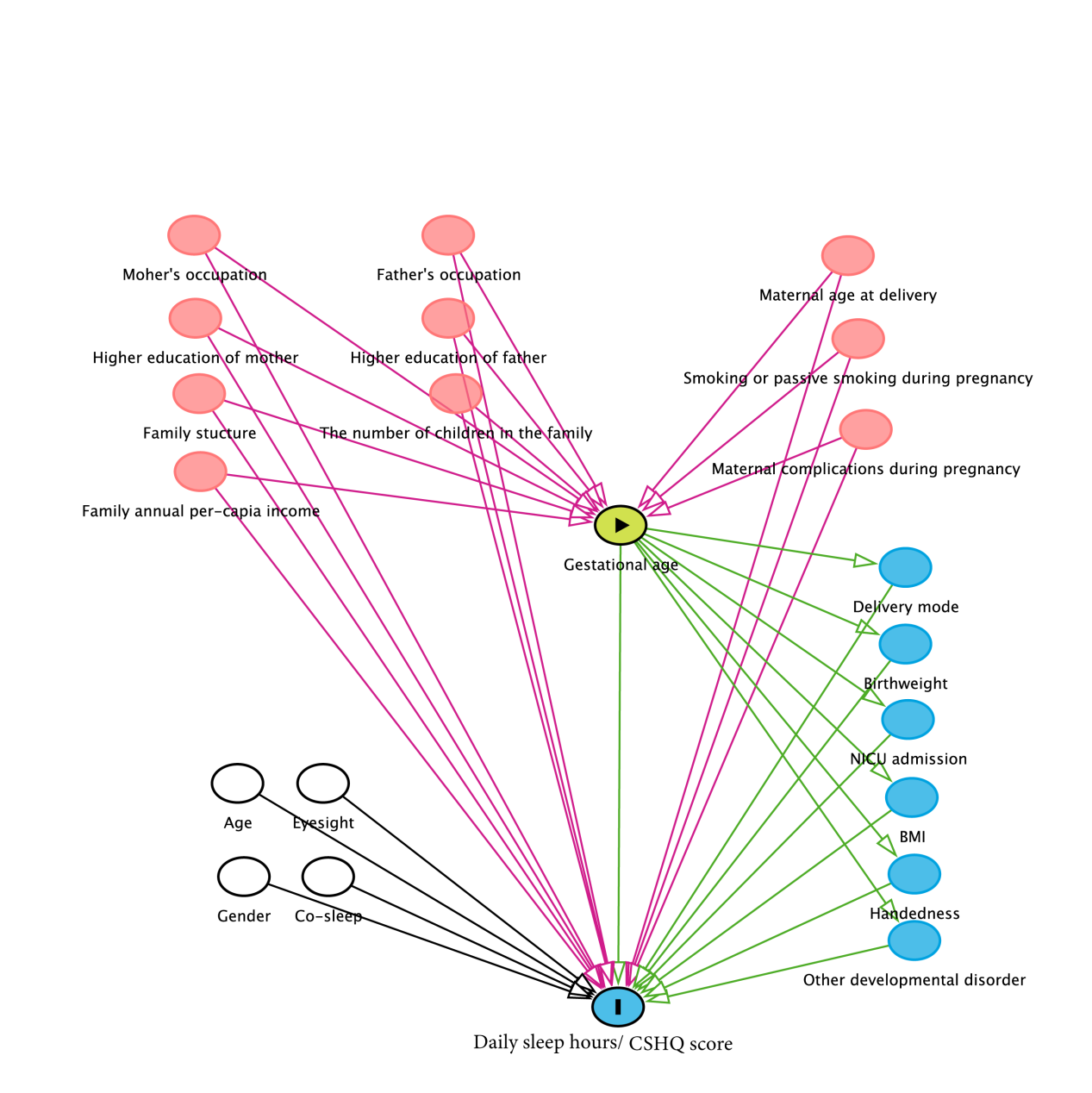


**Competing exposures:**

Age

Gender

Eyesight

Co-sleep

**Confounders:**

**Maternal characteristics:**

Maternal age at delivery

Smoking or passive smoking during pregnancy

Maternal complications during pregnancy

**Family characteristics:**

Mother’s occupation

Father’s occupation

Higher education of mother

Higher education of father

Family structure

The number of children in the family

Family annual per-capita income

**Mediators:**

Delivery mode

Birthweight

NICU admission

BMI

Handedness

Other developmental disorder

Figure S1.

A directed acyclic graph (DAG) describing the relationship between gestational age with daily sleep hours and CSHQ score. Green lines represent paths associated with variables on the causal pathway and were not included in adjusted models.
